# Supplementary material for: Vaccination against SARS-CoV-2 in Haemodialysis Patients: Spike’s Ab Response and the Influence of BMI and Age
Source: Int J Environ Res Public Health. 2022 Aug 15;19(16):10091. doi: 10.3390/ijerph191610091 (PMC9408116; doi:10.3390/ijerph191610091)
Supplement: Supplementary file 1 [file ijerph-19-10091-s001.zip › Supplementary tables/Supplementary Table S1. Anti-spike IgG, according to age and BMI in the vaccinated group at t0.pdf]

Supplementary Table S1. Anti-spike IgG, according to age and BMI in the vaccinated group at t0

				Vaccination group - 3 months (anti-spike IgG)				
				Valid N	Mean	Median	Percentile 25	Percentile 75
Age Group	≤ 60 years	BMI (Kg/m2) Group	< 23	45	3521	898	508	2536
			23 - 28	40	4111	1976	910	3790
			> 28	28	2861	1520	877	3514
	61 - 70 years	BMI (Kg/m2) Group	< 23	19	1669	1754	366	2936
			23 - 28	30	3422	1055	535	2810
			> 28	28	4501	1156	530	2701
	> 70 years	BMI (Kg/m2) Group	< 23	47	4044	838	278	1667
			23 - 28	55	1759	837	351	2075
			> 28	29	1566	979	440	2305

Values are represented as mean, median and Interquartile range (IQR) of anti-spike IgG for age and body mass index (BMI).
