# Supplementary material for: Vaccination against SARS-CoV-2 in Haemodialysis Patients: Spike’s Ab Response and the Influence of BMI and Age
Source: Int J Environ Res Public Health. 2022 Aug 15;19(16):10091. doi: 10.3390/ijerph191610091 (PMC9408116; doi:10.3390/ijerph191610091)
Supplement: Supplementary file 1 [file ijerph-19-10091-s001.zip › Supplementary tables/Supplementary Table S2. Anti-spike IgG, according to age and BMI in the vaccinated group at t1.pdf]

Supplementary Table S2. Anti-spike IgG, according to age and BMI in the vaccinated group at t1

				Vaccination group - 6 months (anti-spike IgG)				
				Valid N	Mean	Median	Percentile 25	Percentile 75
Age Group	≤ 60 years	BMI (Kg/m2) Group	< 23	45	994	429	189	969
			23 - 28	40	1702	718	471	1591
			> 28	28	1151	620	427	1483
	61 - 70 years	BMI (Kg/m2) Group	< 23	19	556	616	183	802
			23 - 28	30	2635	459	198	961
			> 28	28	1816	376	205	802
	> 70 years	BMI (Kg/m2) Group	< 23	47	1738	304	98	776
			23 - 28	55	621	401	128	816
			> 28	29	655	316	172	1210

Values are represented as mean, median and Interquartile range (IQR) of anti-spike IgG for age and body mass index (BMI).
