# Supplementary material for: Vaccination against SARS-CoV-2 in Haemodialysis Patients: Spike’s Ab Response and the Influence of BMI and Age
Source: Int J Environ Res Public Health. 2022 Aug 15;19(16):10091. doi: 10.3390/ijerph191610091 (PMC9408116; doi:10.3390/ijerph191610091)
Supplement: Supplementary file 1 [file ijerph-19-10091-s001.zip › Supplementary tables/Supplementary Table S3. Anti-spike IgG, according to age and BMI in the Sars-CoV-2 recovered (Control group).pdf]

Supplementary Table S3. Anti-spike IgG, according to age and BMI in the Sars-CoV-2 recovered (Control group)

				SARS-CoV-2 positive group (anti-spike IgG)				
				Valid N	Mean	Median	Percentile 25	Percentile 75
Age Group	≤ 60 years	BMI (Kg/m2) Group	< 23	16	3683	1139	287	6292
			23 - 28	20	2013	1822	160	2956
			> 28	15	2899	1769	457	4037
	61 - 70 years	BMI (Kg/m2) Group	< 23	17	7913	1961	1004	8126
			23 - 28	12	5181	1489	938	3188
			> 28	15	6289	1830	1362	4586
	> 70 years	BMI (Kg/m2) Group	< 23	32	8953	1896	1009	7233
			23 - 28	24	9451	1756	515	11979
			> 28	32	8730	2798	1015	8687

Values are represented as mean, median and Interquartile range (IQR) of anti-spike IgG for age and body mass index (BMI).
