# Supplementary material for: Vaccination against SARS-CoV-2 in Haemodialysis Patients: Spike’s Ab Response and the Influence of BMI and Age
Source: Int J Environ Res Public Health. 2022 Aug 15;19(16):10091. doi: 10.3390/ijerph191610091 (PMC9408116; doi:10.3390/ijerph191610091)
Supplement: Supplementary file 1 [file ijerph-19-10091-s001.zip › Supplementary tables/Supplementary Table S4. Comparison of humoral immunity status, according to age and BMI subgroups in the vaccinated group at t0.pdf]

Supplementary Table S4. Comparison of humoral immunity status, according to age and BMI subgroups in the vaccinated group at t0

				Vaccination group - 3 months (anti-spike IgG)				
				Valid N	Mean	Median	Percentile 25	Percentile 75
Age Subgroup	< 70 years	BMI (Kg/m2)	< 30	152	3305	1355	648	3023
			≥ 30	38	4237	1397	609	2853
	≥ 70 years	BMI (Kg/m2)	< 30	116	2714	875	342	2122
			≥ 30	15	1158	738	388	1830

Values are represented as mean, median and Interquartile range (IQR) of anti-spike IgG for age and body mass index (BMI) subgroups. The youngest showed a better humoral response
