# Supplementary material for: Vaccination against SARS-CoV-2 in Haemodialysis Patients: Spike’s Ab Response and the Influence of BMI and Age
Source: Int J Environ Res Public Health. 2022 Aug 15;19(16):10091. doi: 10.3390/ijerph191610091 (PMC9408116; doi:10.3390/ijerph191610091)
Supplement: Supplementary file 1 [file ijerph-19-10091-s001.zip › Supplementary tables/Supplementary Table S5. Comparison of humoral immunity status, according to age and BMI subgroups in the vaccinated group at t1.pdf]

Supplementary Table S5. Comparison of humoral immunity status, according to age and BMI subgroups in the vaccinated group at t1

				Vaccination group - 6 months (anti-spike IgG)				
				Valid N	Mean	Median	Percentile 25	Percentile 75
Age subgroup	< 70 years	BMI (Kg/m2)	< 30	152	1447	518	250	1126
			≥ 30	38	1725	502	221	831
	≥ 70 years	BMI (Kg/m2)	< 30	116	1097	366	124	839
			≥ 30	15	504	291	113	986

Values are represented as mean, median and Interquartile range (IQR) of anti-spike IgG for age and body mass index (BMI) subgroups. The younger ones maintain a better humoral response.
