# Supplementary material for: Vaccination against SARS-CoV-2 in Haemodialysis Patients: Spike’s Ab Response and the Influence of BMI and Age
Source: Int J Environ Res Public Health. 2022 Aug 15;19(16):10091. doi: 10.3390/ijerph191610091 (PMC9408116; doi:10.3390/ijerph191610091)
Supplement: Supplementary file 1 [file ijerph-19-10091-s001.zip › Supplementary tables/Supplementary Table S6. Comparison of humoral immunity status, according to age and BMI subgroups in the Sars-CoV-2 recovered (Control group).pdf]

Supplementary Table S6. Comparison of humoral immunity status, according to age and BMI subgroups in the Sars-CoV-2 recovered (Control group)

				SARS-CoV-2 positive group (anti-spike IgG)				
				Valid N	Mean	Median	Percentile 25	Percentile 75
Age Subgroup	< 70 years	BMI (Kg/m2) Subgroup	< 30	75	4328	1807	728	3722
			≥ 30	20	5455	2114	488	4802
	≥ 70 years	BMI (Kg/m2) Subgroup	< 30	69	9680	1831	856	9803
			≥ 30	19	6566	2869	906	9895

Values are represented as mean, median and Interquartile range (IQR) of anti-spike IgG for age and body mass index (BMI) subgroups. In this group, it was the oldest and heaviest that developed the best humoral response.
